# Editorial: Tailored strategies for lung cancer diagnosis and treatment in special populations

**DOI:** 10.3389/fmed.2026.1782836

**Published:** 2026-02-12

**Authors:** Run-Qi Guo, Natsuo Tomita, Chuan Li, Xin Nie

**Affiliations:** 1 Minimally Invasive Tumor Therapies Center, Beijing Hospital, National Center of Gerontology, Institute of Geriatric Medicine, Chinese Academy of Medical Sciences, Beijing, China; 2 Department of Radiology, Graduate School of Medical Sciences, Nagoya City University, Nagoya, Japan; 3 Department of Thoracic Surgery, Institute of Thoracic Oncology, West China Hospital, Sichuan University, Chengdu, China; 4 Department of Oncology, Beijing Hospital, National Center of Gerontology, Institute of Geriatric Medicine, Chinese Academy of Medical Sciences, Beijing, China

**Keywords:** diagnosis, lung cancer, personalized medicine, special populations, treatment

Lung cancer remains a significant global health challenge, characterized by persistent disparities in diagnosis, treatment, and clinical outcomes among diverse patient populations. To address these inequities, the Research Topic “*Tailored Strategies for Lung Cancer Diagnosis and Treatment in Special Populations*” was initiated. Rather than applying a one-size-fits-all model, this topic emphasizes the importance of precision oncology—integrating patient-specific variables to enhance therapeutic efficacy, reduce toxicity, and improve survival rates, particularly among underrepresented and high-risk populations. The articles featured in this Research Topic represent a comprehensive investigation into tailored strategies, encompassing advancements in diagnostics, therapeutics, prognostic modeling, and healthcare delivery systmes.

## Personalized therapeutic approaches

Several studies underscore the evolving role of immunotherapy and targeted therapy in real-world and specific patient populations. Lackovic et al. demonstrated that pembrolizumab retained its efficacy in advanced non small cell lung cancer (NSCLC) with high PD-L1 expression, even among clinically complex patients, although response rates may differ from those observed in controlled clinical trials. This finding highlighted the importance of real-world evidence in complementing data derived from clinical trials. Similarly, Cao et al. presented a meta-analysis supporting the use of perioperative immunotherapy in NSCLC patients aged ≥65 years, providing age-specific evidence to guide precision treatment in elderly populations.

In the field of targeted therapy, Li, Ma et al. reported a notable case of sustained complete response in HER2-amplified lung adenocarcinoma treated with cadonilimab in combination with disitamab vedotin—a promising therapeutic strategy for a molecular subtype that currecntly lacks standard targeted options. Meanwhile, Huang et al. investigated EGFR-mutated NSCLC with malignant pleural effusion, and suggested that third-generation EGFR-TKIs combined with pleural drainage may represent a rational management approach, while combination with intrathoracic chemotherapy did not yield significant survival benefits in this setting.

## Innovations in diagnosis and prognostication

Advances in non-invasive and artifical intelligence (AI) -enhanced diagnostics approaches are well-illustrated. She et al. demonstrated that spectral CT combined with AI-derived parameters can predict EGFR mutations with moderate accuracy, offering a promising non-invasive alternative to tissue biopsies. Wang, Li, Li et al. further expanded the application of radiomics by developing a CT-based nomogram for predicting brain metastases in patients with ALK-positive lung adenocarcinoma.

Prognostic modeling contiunes to advance personalized adjuvant strategies. Zhao et al. introduced the F-PLR score as a practical biomarker for postoperative prognosis in NSCLC, enhancing risk stratification. Meanwhile, Liu et al. leveraged machine learning to identify early-stage lung cancer patients with a history of breast cancer who may benefit from adjuvant chemotherapy, thereby enabling more tailored therapeutic decisions.

## Special populations

Palecki et al. emphasized the aggressive progression of lung cancer among individuals living with HIV, even in the context of viral suppression-a critical observation that underscores the need for dedicated research and inclusion of this population in clinical trials. Javier et al. highlighted significant disparities in lung cancer screening uptake, with notably lower rates observed in non-White racial/ethnic groups, revealing systemic barriers that impede early detection.

Researches focusing on patients with rare histological subtypes, such as SMARCA4-deficient undifferentiated tumors (Xie et al.) or transformed SCLC (Kawanaka et al.), further highlighted the importance of developing individualized management strategies. The included case report, such as occult lymph nodal metastasis in sub-centimeter lung cancers (Wang, Li, Shi et al.) or adenocarcinoma and squamous cell carcinoma in the same lobe with adenocarcinoma metastasis in the lymph nodes (Li, Zou et al.), exemplified the complex diagnostic and therapeutic challenges encountered in clinical practice.

## Supportive care

Additonally, the topic acknowledges the importance of supportive care. Cai et al. systematically evaluated non-pharmacological interventions for constipation in lung cancer patients, recommending acupoint therapy combined with massage-an integrative approach that aligns with patient-centered care.

## Moving forward: from evidence to equity

The findings of this Research Topic underscore that effective, tailored strategies must extend beyond tumor biology to encompass the holistic patient profile, including age, comorbidities, genetic background, lifestyle, and social context. Machine learning and AI show considerable promise in integrating these multifaceted variables into actionable clinical tools, as evidenced accross multiple studies. However, given the persistence of disparities in screening and unequal access to care, translational advances must be accompanied by deliberate, equity-focused initiatives. These include broadening trial eligibility criteria, validating biomarkers across diverse ethnic populations, implementing culturally competent care models, and actively addressing structural barriers to both screening and treatment.

We thank all authors for their valuable contributions, which collectively advance the paradigm of precision oncology in lung cancer. It is our hope that this Research Topic will inspire further interdisciplinary research, foster collaborative care models, and ultimately contribute to more equitable and effective lung cancer management for all patients, especially those in special populations.

